# Performance Metric Analysis for a Jamming Detection Mechanism under Collaborative and Cooperative Schemes in Industrial Wireless Sensor Networks

**DOI:** 10.3390/s22010178

**Published:** 2021-12-28

**Authors:** Alejandro Cortés-Leal, Carolina Del-Valle-Soto, Cesar Cardenas, Leonardo J. Valdivia, Jose Alberto Del Puerto-Flores

**Affiliations:** 1 Facultad de Ingeniería, Universidad Panamericana, Álvaro del Portillo 49, Zapopan 45010, Jalisco, Mexico; 0005358@up.edu.mx (A.C.-L.); lvaldivia@up.edu.mx (L.J.V.); jpuerto@up.edu.mx (J.A.D.P.-F.); 2 Tecnologico de Monterrey, Universidad Internacional de La Rioja (UNIR) en Mexico, Mexico City 03600, Mexico; cesarraul.cardenas@unir.net

**Keywords:** Industrial Wireless Sensor Networks, jamming, Industrial Internet of Things (IIoT), cooperative communication, security for IIoT

## Abstract

The emergence of Industry 4.0 technologies, such as the Internet of Things (IoT) and Wireless Sensor Networks (WSN), has prompted a reconsideration of methodologies for network security as well as reducing operation and maintenance costs, especially at the physical layer, where the energy consumption plays an important role. This article demonstrates through simulations and experiments that, while the cooperative scheme is more efficient when a WSN is at normal operating conditions, the collaborative scheme offers more enhanced protection against the aggressiveness of jamming in the performance metrics, thus making it safer, reducing operation and maintenance costs and laying the foundations for jamming mitigation. This document additionally offers an algorithm to detect jamming in real time. Firstly, it examines the characteristics and damages caused by the type of aggressor. Secondly, it reflects on the natural immunity of the WSN (which depends on its node density and a cooperative or collaborative configuration). Finally, it considers the performance metrics, especially those that impact energy consumption during transmission.

## 1. Introduction

The growing trend in smart factories encompasses both the IoT and WSN technologies, which impose new challenges for industrial safety, operation, and maintenance. Several technologies converge at the IoT, such as WSNs, real-time computing, embedded systems, and actuators [1]. By 2022, circa 62% of the world’s connected devices will adopt IoT technologies [2]. In 2020, there were 21 billion IoT devices, a number that may double by 2025 [3]. This accelerated growth entails new challenges.

One challenge consists in improving *security*. In the first half of 2019, attacks on IoT-based devices increased by more than 300% [3]. One of the most aggressive attacks on energy consumption is jamming, which distorts the sending and receiving frequencies using heavy noise levels [4]. These attacks on Industrial Wireless Sensor Networks (IWSN) seek to deteriorate communication between network nodes by issuing proactive and reactive signals without following specific protocols [5]. Jamming attacks must be detected and mitigated quickly to maintain security and reduce energy consumption. The optimal maintenance of the data transmission by the sensors that are part of a WSN is imperative for optimizing the devices used for maintaining industrial machines, automating home appliances, monitoring healthcare, livestock, and crops, observing traffic, public transport, or pollution at a smart city, as well as for detecting potential fires and controlling energy consumption. Databases must receive secure information for decision-making. The early detection of jamming plays a relevant role to secure the data that the sensors collect and store.

Another challenge is keeping low operating and maintenance *costs*. According to Dargie, W. [6] and Singal, T. [7], applications with WSNs provide higher reliability, availability, resilience, ease of installation, and coverage than wired networks. The most common metrics that impact the reduction of maintenance costs in a WSN are reliability and availability. *Reliability* indicates the probability of performing a required function or operation under certain conditions within given time intervals [8]. A high percentage of the Packet Delivery Ratio (PDR) metric indicates high reliability as the network takes little time to discover routes, has few retransmissions, and has a low hop count. Reliability is achieved by having available routes, maintaining a low hop count, quickly transmitting the packets, and maintaining a routing table that handles the proper packet traffic. On the other hand, *availability* means the ability of a system to be in a state in which it can execute an operation or function at a given moment or within a given time interval [8]. Reducing the delay times in the hops obtains the availability of WSN for a faster diagnosis and recovery time and keeping valid routes for as long as possible, consequently achieving fewer retransmissions, consuming less energy in sending packages, and reducing the uptime of the sensors. A good level of the Received Signal Strength Indicator (RSSI) metric and the Link Quality Indicator (LQI) indicate that there would be greater availability, which is evident in a rapid diagnosis and recovery of the network from a jamming attack.

Realizing the importance of having proposals to increase WSN security, we have generated the following question: *Which configuration helps more to protect a WSN from jamming?*

### Motivation

Our motivation arises to seek measures to quickly detect and mitigate jamming, as it is one of the most aggressive attacks on energy consumption. Given that cybercrime against companies amounts to a loss of nearly USD 6 trillion per year, and it could cost the world USD 10.5 trillion annually by 2025 [9], the investment by companies to solve jamming has a prospect of growth of 7.9% at a Compound Annual Growth Rate (CAGR) from 2020 to 2025 [10]. We aspire to protect the WSN from jamming by monitoring the values of the WSN metrics. The performance metrics are altered in different ways according to the type of jamming (constant, deceptive, random, and reactive) and the scheme that the network uses to communicate, which can be cooperative (network-centric) or collaborative (node-centric).

All of the above factors motivate us to propose an algorithm to detect jamming in real time, considering the above-mentioned two factors, especially in *industrial environments*. Since the behavior of the performance metrics varies depending on the different *node densities*, this article carries out two simulations to provide proper data history for the algorithm configuration. An experiment on a WSN with the same characteristics as an IWSN validates an algorithm that recognizes whether the network is under attack and reveals both the kind of jamming which is attacking the network. This learned information serves to improve the response to future attacks. With this algorithm, the foundations are laid for future works for the mitigation of jamming, its impact on energy consumption, and the economic benefits it entails to the factory.

According to the research question, which arises from a need that the industry has, the objective of this work is to propose a jamming detection mechanism for IWSNs based on the performance metrics analysis under collaborative and cooperative schemes. It is desired that the proposed mechanism can be used as a starting point in future work to propose a jamming mitigation method that increases the availability and reliability of the network.

The structure of this article is as follows: Section 2 describes the related works on jamming detection techniques, metrics, density of nodes and network schemes. Section 3 proposes a jamming detection mechanism and describes the simulation and experiment parameters. Section 4 analyzes the benefits that the collaborative WSN offers during a detected jamming attack on the network. Section 5 discusses the results. Finally, Section 6 concludes this article by making suggestions for future work.

## 2. Related Works

To obtain an answer to the research question, related works have been sought on the types of jamming, techniques for detecting jamming and WSN performance metrics, as well as node densities and cooperative and collaborative schemes.

Jamming is one of the most effective denial of service (DoS) attacks; the attacker prevents legitimate data from reaching its target and causes packets to collide, so legitimate packets cannot be delivered through channels [4]. A classification of the existing types of jamming is presented in Table 1.

While *elementary proactive jamming* sends jamming interfering signals in a network whether the data communication is there or not, *reactive jamming* senses the network in active state and ongoing communication and then it initializes the sending of jam signals. Reactive jammers use more energy than active jammers as they are always monitoring the network, going to an active state every time there is a transmission on the channel. On the other hand, advanced *smart hybrid jamming* [11] can be proactive, reactive or hybrid. This attack hinders the communication bandwidth in an important part of the network, adding energy in certain strategic places; however, it could be *function-specific* [11], having a programmed function that attacks a single channel or multiple channels simultaneously, maximizing the jamming throughput irrespective of the energy usage. It can change to another channel according to their specific functionality.

The most common attacks in the industrial environment are elemental elements, which in IWSNs are sometimes confused with the noise generated by the machines in the production process. The first elementary type of jammer is the *constant* [4,12,13,14], which is achieved by sending bits continuously to the network without an established protocol to saturate the transmission channel, and so that the legitimate nodes cannot engage in communication correctly, Carrier Sense Multiple Access with Collision Avoidance (CSMA/CA) protocol is not validated as random bits are constantly sent. Nodes receive corrupted packets and the processor works more, which leads to greater battery consumption. The second is *deceptive* jammer [4,14,15], which is simulated by sending packets continuously, but they are legitimate packets and no longer random, causing legitimate nodes to always be listening, and with a lot of traffic, they cannot change from receive state to send state. Since they have the appearance of a legitimate transmission, their impersonation is more difficult to distinguish. The third is *random* jammer [4,12,13,14], which, being also active, tries to block communication and can be simulated if interference periods alternate with sleep state periods. When it emits interference, it does so randomly, behaving like a constant jammer or as a deceptive jammer, while when it switches to the sleep state it acts as a reactive jammer and stops consuming energy considerably, decreasing data processing and increasing battery life. Finally, *reactive* jammer [4,12,13,14] is the most difficult to detect. It works a little differently from the previous three types of jamming since they consume less energy when listening to the communication channel and, when it detects a transmission, it emits a small radio signal, which is enough to cause collisions but not to be detected. This mode of jamming presents a greater energy consumption, but they are more effective in their objective since, in the short time in which they act, they can cause a lot of damage.

Regarding the impact on the economy, Sadiki, S. et al. [16] explore the economic impact of the WSN in the industry, showing through a simulation the effect of applying the WSN in the metrics of industrial maintenance. Improving the latency and energy performance [17] of a WSN inside a factory can positively impact the predictive maintenance program costs as the WSN provides the data acquisition. According to Misra, S. et al. [14], energy can be obtained by multiplying the squared value of the voltage drop of the sensor node battery by the time, and dividing the result by the average electrical load at the node. Another way to obtain it is to add the reading of the different energy consumption generated in the node [18,19]:

(1) $$\begin{array}{c} Energy=E_{M}+E_{S}+E_{SD}+E_{CSMA}+E_{SW1}+E_{SW2}+E_{T_{x}}+E_{R_{x}}. \end{array}$$

where $E_{M}$ is the energy from micro-controller unit (MCU) running on 32-MHz clock, $E_{S}$ is the energy in start-up mode, $E_{SD}$ refers to the energy in power down sleep mode, $E_{CSMA}$ is the energy consumed by CSMA/CA algorithm, $E_{T_{x}}$ refers to the energy consumed by a node in transmission mode, $E_{R_{x}}$ is the energy consumed by a node in reception mode, $E_{SW1}$ refers to the switching energy from *R_x_* to *T_x_* and $E_{SW2}$ to the switching energy from *T_x_* to *R_x_*. Other performance metrics used in this study are [17] the *PDR* which is the ratio of the number of packets successfully sent by the source over the total number of packets transmitted at the source [20]. It can be expressed as:

(2) $$\begin{array}{c} PDR=\left[\frac{P_{SS}}{P_{T}}\right] \end{array}$$

where $P_{SS}$ is the number of packets successfully sent by the source and $P_{T}$ is the total number of packets transmitted at the source. To detect jamming, PDR must be complemented with other metrics, since its variation could also be related to other factors such as imperfect connections, collisions or neighbor node failures [21]. Another used metric is the Link Quality Indicator *LQI*, which indicates if the path that a message takes to propagate in a mesh is good in comparison to other paths. An LQI level must be up to 50 to be considered as acceptable, and a value of 120 is very good [22]. The *RSSI* is the signal strength distribution and can be estimated as [23,24]:

(3) $$\begin{array}{c} T_{RE}=T_{SE}\cdot G_{T}\cdot G_{R}\cdot \left[\frac{\alpha }{4\pi d}\right] \end{array}$$

where $T_{RE}$ refers to the remaining energy received at the receiver end, $T_{SE}$ refers to the transmission energy of the sender, $G_{T}$ is the transmitter gain, $G_{R}$ is the receiver gain, α is the wavelength and *d* is the distance between the sender and the receiver. With the previous result, the RSSI can be obtained as:

(4) $$\begin{array}{c} RSSI=10\cdot log\left[\frac{T_{RE}}{R_{e}\cdot f_{P}}\right] \end{array}$$

where $R_{e}\cdot f_{P}$ is the reference energy, experimentally equivalent to 1mW. RSSI impacts LQI; a good RSSI implies a greater probability of finding available routes, reducing transmission times and also impacting the decrease in energy consumption. It is better to use the LQI as a link estimator than the RSSI for high stress applications such as underground mines [25].

In other works, energy savings have been sought in the WSN by putting nodes to the sleep mode, which implies increasing the duration of a node operation cycle, and the cycle includes operations that affect energy consumption. The useful life of the node batteries is highly dependent on energy savings [26]. According to Del-Valle-Soto, C. et al. [18], the energy consumption is reduced by putting the node in sleep mode since the the node consumes more power when in transmission mode than when in sleep mode. However, the problem of jamming attacks can be approached in different ways. Osanaiye, O. et al. [20] have used an exponentially weighted moving average method to detect jamming using the packet inter-arrival time of the packets received from the sensor nodes. The authors in study [27] propose a technique based on clustering approach and timestamp which made a contribution to the grouping of sensor nodes and the timestamp calculated from one node to another node. A PDR and RSSI metrics are used by Vijayakumar, K. et al. [28] to detect jamming with two methods: fuzzy inference system (FIS) and adaptive neuro-fuzzy inference system (ANFIS). On the other hand, Kanagasabapathy, P.M. et al. [29] propose two approaches for cluster-based WSN. The first is based on detecting the level of maliciousness of the nodes using a certification module for the defense of the network. The second is based on monitoring, using fuzzy logic to discover which nodes are being affected by a jammer. On the other hand, Corral-Molina, C. [30] proposes a security method against the jammer based on the Bit Error Rate (BER) and Signal to Noise Ratio (SNR) metrics. The above provides greater readability of the message in the legitimate receiver and finds an energy distribution that favors the transmission of packets.

There are several ways to detect the presence of intentional jamming interference in a WSN. An overview of detection techniques is presented in Table 2.

We become aware of the presence of jamming because the WSN begins to behave differently, acquiring readings altered to normal, thus showing the influence of each type of jamming with the behavior of each of the performance metrics. In this work, these anomalies in the network performance metrics are referred as *symptoms*. The symptoms are manifestations that indicate that the state of health of a WSN is not in normal conditions, such as a very low level of PDR, or a very high level of energy consumed. In Table 3, we present some of these relationships between jamming and metrics. The variations of *increase*, *decrease* or *oscillation* of the metrics are with respect to the values of these present in the steady state. It is observed that constant and deceptive jammers in general cause a greater decrease in the PDR and a greater increase in the negative value of the RSSI. As there are fewer packets received and less signal strength, the search for routes with better quality becomes greater, consuming more time and energy in processing the information for forwarding. The relation between the above metrics are used in many ways to detect and mitigate jamming attacks. If, for example, the routing table of a node increases considerably, its processor must work more and consume more energy, having less time to react to any contingency.

Resilience is the ability to recover in a short time from a communication failure, reducing the transmission delays by minimizing the number of hops. A resilient WSN maintains good RSSI levels [41]. RSSI values are in dBm. A level close to 0 dBm represents a good signal level, while a signal level close to −110 dBm indicates that the signal is very poor, which may be due to environmental conditions, the presence of jamming, low-density network nodes, etc. [12]. On the other hand, a good PDR measurement (close to 100%) allows us to take better advantage of the processor memory, which will heat less by not having to process more information unnecessarily [14]. The released heat comes from the electrical energy and impacts less battery life.

In the detection of the existence of jamming, a normal value of one can be taken for the SNR metric, since this parameter is the relationship between the signal to be communicated with the noise signal. A less than one value would mean that the noise is greater than the transmitted signal and would make it opaque [20]. Closely linked to the SNR is the BER metric [30], which gets a high value only when the SNR is low [42]. When the noise is greater than the transmitted signal, the SNR metric is less than one, and this has an exponential impact on Bit Error Rate (BER) metric. The reduction of retransmissions, as well as hops, is important in a network to ensure that a greater number of packets reach their destination. Due to the above, the BPR metric can complement the SNR and the BER in the detection of jamming [20].

A strong signal is less likely to have errors than a weak one. If an error increases with SNR, it is due to noise, but this noise could be due to unintended interference from, for example, motors and actuator on a machine in a factory. Another scenario of metric interdependence is observed: when the LQI in the network is increased, energy consumption and waste are decreased, as well as the number of collisions, interferences and transmission time. On the other hand, a better quality in the link would increase the PDR (%), since the packets would arrive intact at their destination, reducing the processing of data in memory and guaranteeing that all the routes in the routing table are valid.

The result of the behavior of the different metrics can be analyzed to grant a classification regarding the Quality of Service (QoS) level. As an example, the QoS parameter metrics to evaluate IEEE 802.15.4 protocol performance in a star topology are presented by Mohanty, S. [43], taking into consideration metrics such as PDR, ECA, network lifetime and percentage of time in sleep mode; moreover, a general definition of QoS is put forth. QoS could be measured for the physical (hardware) layer in terms of PDR, RSSI, ECA and Bad Packet Ratio (BPR).

The change in the levels of performance metrics depends on various factors. In an IWSN, the interference experienced can be caused by *unintentional* radiant sources or by *intentional* noise, causing in both cases the deterioration of communication on the network and a state of vulnerability in certain coverage areas [44]. Some examples of *broadband* interferences include unintentional transmissions coming from motors, inverters, power circuits, electric switches and contacts, electrostatic devices, ignition systems, voltage regulators, lighting electromagnetic pulses, pulse generators, thermostats, welding machines, frequency converters, etc. On the other hand, *narrowband* interferences consist in *intentional* transmissions coming from cell phones, TVs, radios, line hums, signal generators, local oscillators, test equipment, microwaves, ultrasonic equipment, electronic ballasts, medical equipment, microprocessors, high-frequency generators, etc.

**Figure 1:**
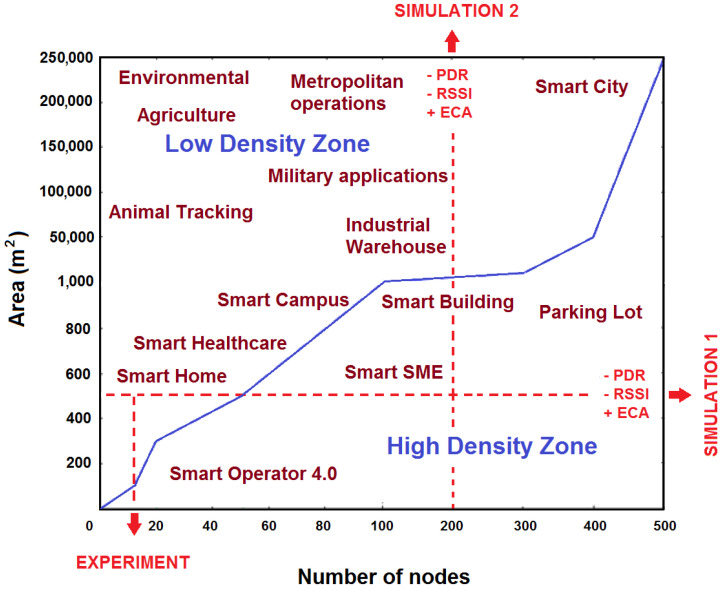
WSN applications based on node density.

Other works have focused on relating the *density* of the nodes of a network with the damage that a jamming attack produces to the performance metrics. Al-Shaihk [45] uses node density and initial node power as evaluation criteria to find the *survivability* (the ability to provide basic services after an interference attack) of a network after an interference attack. If the node density and the initial power increase, the survivability increases, manifesting itself in an increase in the PDR measurement. Secondly, Hamidzadeh, J. [46] mentions that if a cluster-based WSN size is high and the density of the cluster is high, it leads to wasting more energy of cluster heads that are far from the base station. Finally, Deepa Kalaimani et al. [47] propose an energy-proficient clustering approach that performs better in terms of ECA, PDR and network lifetime. We define node *density* as the number of network nodes present in a certain geographic area. WSNs have a large number of practical applications [48,49], ranging from places with a very low density of nodes per area, such as environmental monitoring stations, agricultural care, animal tracking and some military applications, to WSNs with a high density such as applications within hospitals, buildings and smart factories. An extra effort was made to know for different contexts the types of practical applications that exist. Some WSN applications for various network densities are suggested in Figure 1.

The upper left area represents networks with a low density, because there are few nodes in a very large area, and the lower right area represents networks with a high density, that is, with many nodes in a small area. The blue line represents the boundary between the zone of high and low node density. According to Güngör, V. Ç. [50], a high density of nodes within a geographic approach can improve network connectivity, causing an increase in the reliability of deliveries, but entails an increase in latency due to the use of non-optimal forwarding routes. With this work, we also want to know which is the best network scheme to protect the WSN against a jamming attack, so we want to explore the behavior of the network before different configurations, which include the number of nodes, the geographic area where the WSN is installed, if the processing scheme is cooperative or collaborative, etc.

**Figure 2:**
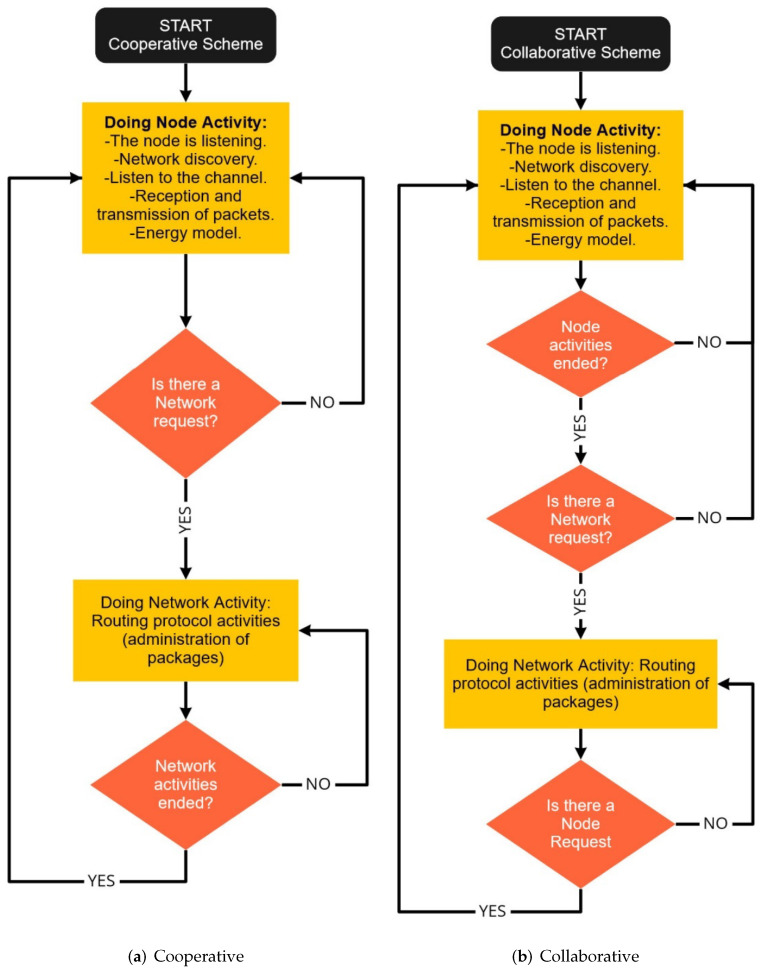
Schemes for WSNs.

In a *cooperative* scheme [48,51], the node is more committed to the other nodes since it shares its resources by following strict rules and protocols in order to fulfill certain assigned functions that help the entire network. The node must first cover the priorities of the network and then its own. On the other hand, in the *collaborative* scheme [48,51], the wireless element shares its resources only when it is available and its operation is not compromised and only if its priorities are covered first. As seen in Figure 2a, the cooperative scheme gives preference to the network since, at any request from the network, it stops performing its activity to perform the activity that the network asks. On the other hand, Figure 2b presents the collaborative scheme, which consists of a synchronous scan of the nodes, in which it needs to finish the node’s task in order to attend a request for the network. The two types of schemes are understood as mutually exclusive, so it is convenient to find the possible uses that can be given to each of the two classes, either in *normal operating conditions* or in a state of *jamming attack*.

It has been shown in [51] that for a network of 20 nodes, located within an area of 500 m${}^{2}$, the cooperative scheme provides better results than the collaborative one when there are normal network operating conditions. In order to relate the different types of jamming and the collaborative and cooperative schemes, an experiment will be carried out in a similar area and condition to determine when it is convenient to use one scheme or the other.

In the following section, the detection algorithm is proposed. Furthermore, two simulations and the real experiment indicated in Figure 1 are designed.

## 3. Materials and Methods

**Figure 3:**
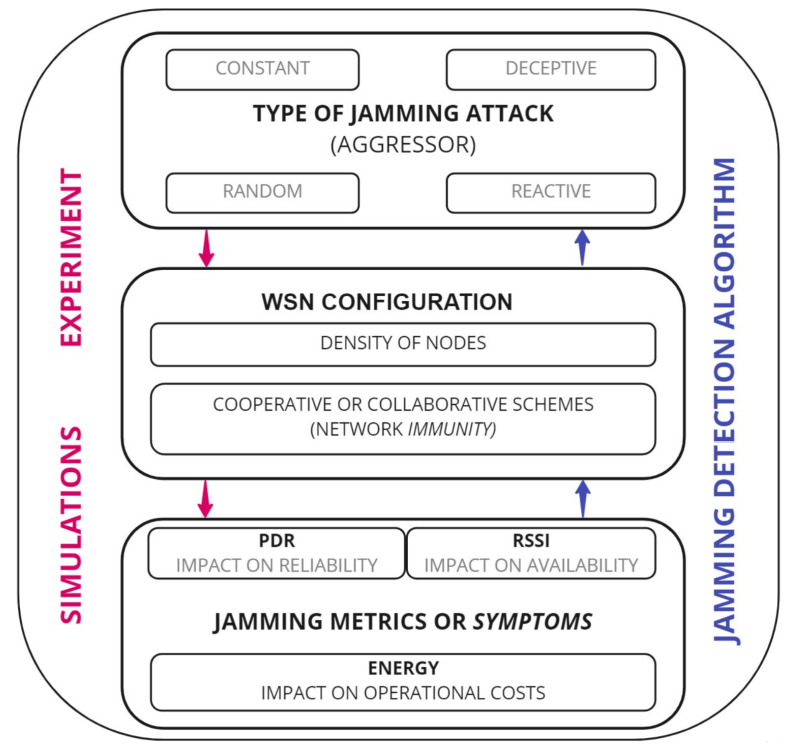
Proposed detection mechanism.

The *hypothesis* of this work is that a WSN can be protected from jamming using a mechanism for its detection, which is based on performance metrics and uses cooperative and collaborative schemes for any density of nodes. To accept or reject this hypothesis, we have designed a *jamming detection mechanism*. The mechanism is showed in Figure 3 and is carried out in two directions. First, the *down arrows* indicate the way *simulations* and *experiments* with jammers bring information on how jamming attacks impact performance metrics. Second, the *up arrows* indicate the way in which a *jamming detection algorithm* obtains the type of jammer that is attacking the network.

The jamming detection mechanism includes the following elements for simulations and the experiment:

- *WSN configuration*, which takes into account the *density* of nodes, consists of the number of nodes and the geographic area in which a WSN is being applied, and the *immunity* of the WSN, which refers to how much the network configuration protects the network from attacks. The simulations and the experiment are carried out under cooperative and collaborative schemes.
- The selected jamming metrics or *symptoms* which are outputs or *dependent variables* that the network presents in a steady state. The selected metrics (PDR, RSSI and Energy) were described in Section 2 and are provided by the results of the simulations and the experiment.
- The *aggressor*, which is the input or independent variable as it refers to the type of jamming attack. The four elementary types of jamming attacks shown in Section 2 are simulated, which are the *aggressors* who attack the network, affecting its performance metrics.

The *jamming detection algorithm* will follow the opposite route to simulations and the experiment. The obtained values for the performance metrics in the simulations and the experiment are integrated into a data history to detect the type of jamming. The algorithm for jamming detection is presented below.

### 3.1. Jamming Detection Algorithm

**Figure 4:**
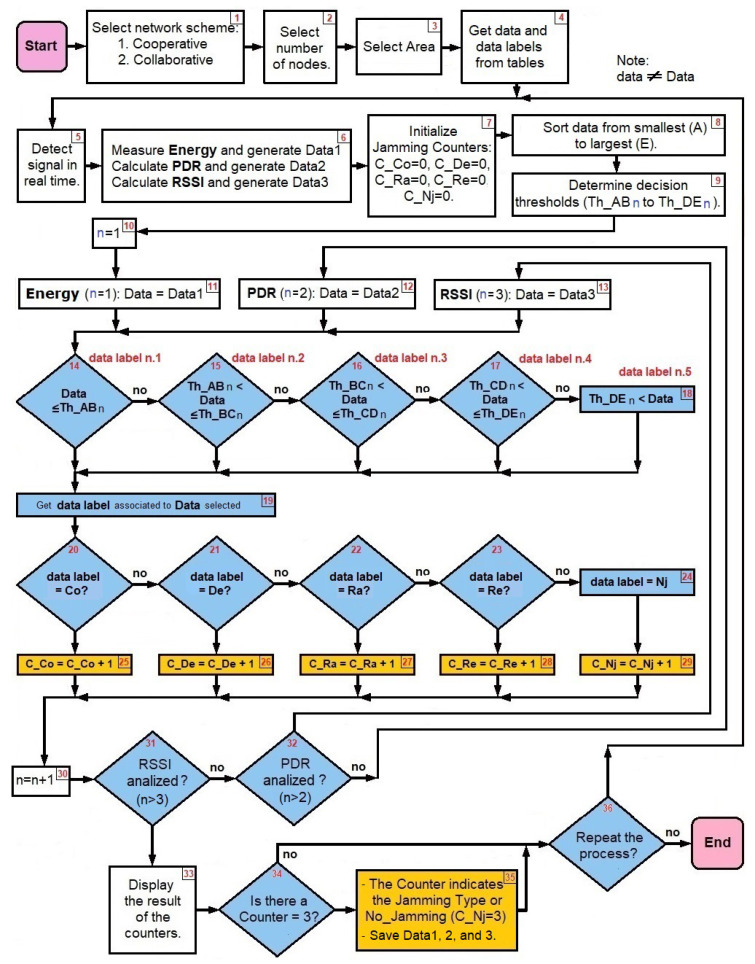
Proposed jamming detection algorithm.

The *jamming detection algorithm* is based on performance metrics and uses as input the cooperative and collaborative schemes as well as the density of nodes, as seen in Figure 4. It is a practical method that consists of a continuous calculation of the PDR and RSSI metrics, which are complemented with Energy to distinguish the type of interference that is present in the network.

From among all the metrics presented in Table 3, we chose only three representative metrics that were sufficient to exemplify the performance of a sensor. The selected metrics are RSSI, PDR and Energy. The proposed mechanism uses these metrics according to the actual performance of the sensors that we use in the experiment. First, the sniffer of the sensors with which the experiment was carried out makes it possible to easily measure the RSSI, from the MAC layer. On the other hand, the PDR, from the network layer, is an easy metric to count with any sensor brand. Finally, the Energy, in all the layers, is programmed with a model that has already been tested in other research works. This makes the model more representative, more random and a bit more reliable.

The algorithm begins by requesting as inputs the network scheme, the number of nodes and the area, taking into account that the counters of the four types of jamming and *no jamming* are at zero. Next, a database provides the data tables obtained with the simulations and the experiment. Next, the signal from WSN in real time is detected, PDR and RSSI are calculated and Energy is measured. All jamming counters are then reset. All the data from tables is organized from smallest (A) to largest (E). The decision *thresholds* are then determined by *taking the averages* of each pair of data groups already ordered from lowest to highest. The process is carried out following the statistical technique of *moving average* in which the average of the *m* elements contained in a sample is taken (in our algorithm *m* = 2) causing an aliasing effect.

Figure 4 can be written as a pseudo-code (see Algorithms 1–4). The energy comparisons are made first (when *n* = 1), taking the energy data in real time and seeing between which *decision thresholds* that data falls, and consequently we associate the *data label* to the *data selected*. A similar process is carried out with the PDR (when *n* = 2) and the RSSI (when *n* = 3), taking into consideration that the order of the data from lowest to highest does not always come from the same type of jamming.

Once the iterations with the three performance metrics are completed, the *data label* ($C_{O}$, $D_{E}$, $R_{A}$, $R_{E}$, $N_{J}$) is associated to the *data selected* (real-time Energy, PDR and RSSI values), transferring their resulting values to the corresponding jamming type counters, and if the result obtained is equal to three, it indicates the presence of that kind of jamming. In the event that the counter value is one or two, the process must be repeated with other real-time measured data. In the absence of jamming attacks, the *no jamming*$\left(C_{N_{J}}\right)$ counter will have a value of three. According to the measurements of all the metrics mentioned above, an alarm of the presence of jamming would be produced, indicating what type of jamming the network is facing, so that an algorithm for mitigation can be implemented immediately. In addition, when a type of jamming has been detected, the real-time information that originated this detection can be stored (Step 35) for reference wherein future artificial intelligence or machine learning could be used to improve the algorithm. Through this algorithm, the aggressiveness suffered by the network from jamming can be labeled and the bases for its mitigation can be laid.
**Algorithm 1:** Proposed jamming detection algorithm for collaborative and cooperative WSN. Part 1.
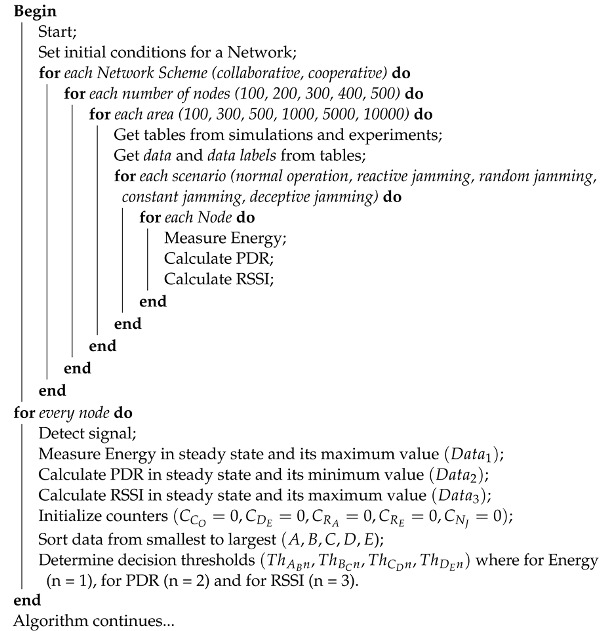

**Algorithm 2:** Proposed jamming detection algorithm for collaborative and cooperative WSN. Part 2.
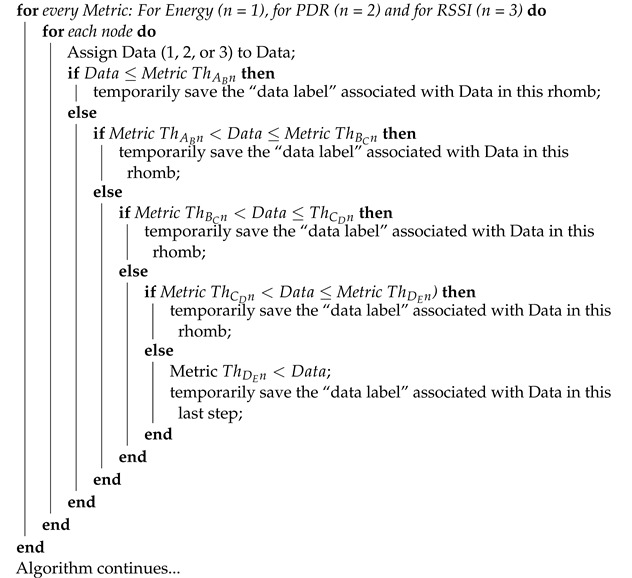

**Algorithm 3:** Proposed jamming detection algorithm for collaborative and cooperative WSN. Part 3.
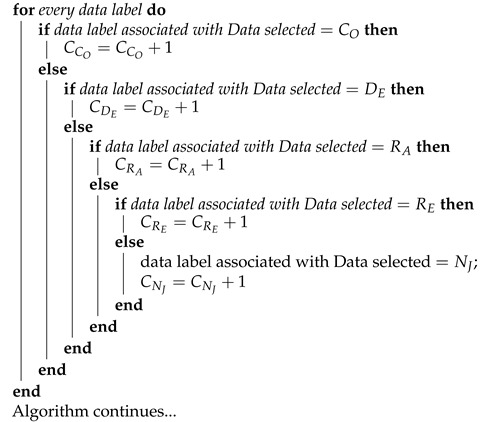

**Algorithm 4:** Proposed jamming detection algorithm for collaborative and cooperative WSN. Part 4.
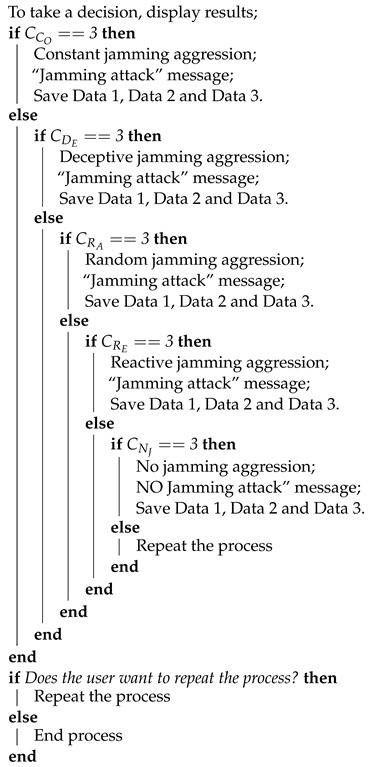


By implementing early detection of jamming interference in different scenarios, such as factories or smart buildings, and selecting a collaborative or cooperative approach, effective communication between sensors and actuators can be be improved.

In the next section, as part of the jamming detection mechanism, the simulation and experimentation parameters are presented.

### 3.2. Simulations

To understand how aggressive the types of jamming would be in different WSN applications, two simulations were run under different node densities and cooperative and collaborative schemes.

Simulation 1: The simulation of the network metrics will be carried out taking the following number of nodes: 50, 100, 200, 300, 400 and 500, with a fixed area of 500 m${}^{2}$.

Simulation 2: The simulation of the network metrics will be carried out taking the following areas (m${}^{2}$): 100, 300, 500, 1000, 5000 and 10,000, with a fixed number of nodes of 200.

This configuration has been chosen to explore possible network scenarios found in an industrial environment [50,52], where hundreds or even thousands of nodes distributed in the geographic space of the factory can coexist. The simulations and the experiment also seek to validate whether in high or low density networks it is convenient to use a collaborative or cooperative scheme.

To distinguish the types of jammer, the simulation takes into account the following send intervals and types of packets [53]: while the *constant* jammer sends *random* bits every millisecond, the *deceptive* jammer sends *regular* bits every millisecond. For its part, the *random* jammer sends both random and regular bits in a time interval between 1 and 100 milliseconds and then goes into sleep mode. Finally, the *reactive* jammer sends bits of how long the legitimate transmission lasts on the communication channel. If it is an RTS/CTS reactive jammer, it is sent during RTS/CTS, and if it is a Data/ACK jammer, it is sent during Data/ACK.

To simulate the cooperative and collaborative behavior of the nodes, a change was made in the task processing algorithm. The *cooperative* scheme is based on the *interrupt method*, which under normal operating conditions offers better micro-controller performance [54]. On the other hand, the *collaborative* scheme is based on the *polling method* which consists in synchronous scanning of the nodes where the micro-controller works continuously, spending more Central Processing Unit (CPU) cycles and being more likely to lose data and consume more power. Because it consumes less energy under normal conditions, the interrupt method is considered better than polling.

To carry out the simulations, the *Configurable Multi-Layer WSN* (CML-WSN) has been used. It is an event-driven simulator and is programmed in C++ language. The parameters offered by the IEEE 802.15.4 standard were taken, because they are used in intelligent industrial environments by IWSN. In the simulations, the distribution of the arrival of packages follows a *poisson distribution*. The simulator accepts several input settings for the physical, MAC and network layers, and it has already been used and tested in other research works [18]. The simulator has an *energy model* to support any sensor specification, and it is useful to explore prototypes. Its architecture consists first in the main *inputs*, which are the number of nodes, the energy model and some data such as maximum hop number, node coverage range, fixed or random topology, data length, data rate, the maximum number of neighbors, process time, transmission time, propagation time, stand out. sampling period, etc. In the second place, the simulator has a *scheduler*, which is an entity with a global vision of the network and which is in charge of managing and controlling the events. The main *events* that it manages are the hello packets, the CSMA/CA algorithm, the packages, the requests, and the statistic timers. In addition, it can record all events during the simulation. The main *actions* that it records are the following: turn on all nodes; turn off nodes; turn on a specific node; look for routes in routing tables; check if an ACK is received; send HELLO packet to make the topology, routing protocol and traffic packets; CSMA/CA algorithm; obtain times to record simulation information; etc. Third, the *outputs* provided by the simulator are the time stamps, general statistics, energy statistics, routing tables and the connectivity matrix.

The sensors will interact within the network with the collaborative scheme and then with the cooperative approach, and the behavior of the PDR, RSSI and energy metrics of the network will be monitored under these paradigms. In this work, the energy in active mode of a sensor is used since we want to show only the main tasks of the sensor in the network: those that demand the most energy or that have the greatest dominance in energy consumption. In order to simulate the possible jamming in IWSN, the physical, MAC and network layer parameters need to be declared. As seen in Table 4, the sensor nodes send packets with a rate of 1% to 4% to a coordinator (sink node); the transmission power used is 0 decibel milliwatts (dBm) for active transmission mode and the receiving power sensitivity threshold is −85 dBm. Because of the noisy environment, the network uses the World Wide Industrial, Scientific and Medical operational frequency band of 2.4 GHz, and uses the worldwide parameter of maximum bit rate declared as 250 kbps. The WSN has been configured inside a MAC layer under the CSMA/CA Protocol, and is used with a maximum of five CSMA retries and three retransmissions per packet. While transmission happens, the nodes wait for the channel to be idle to a new start transmission.

The simulator calculates the energy consumption using the model shown in Equation (1). This energy consumption model takes into account all the activities or tasks that a node executes when it is in active mode. The electrical power is calculated, which is the product of the *voltage* to which the node is connected and the *current* that flows through it. As each activity of the node lasts a certain specific *time*, multiplying the electrical power by time gives the energy in *joules*. Table 5 presents the information of the energy model used for the simulations and experiment [58].

As a result of the simulations carried out with the parameters described above, it is intended that the first part of the jamming detection mechanism has data to be used by the proposed algorithm.

### 3.3. Experiment

**Figure 5:**
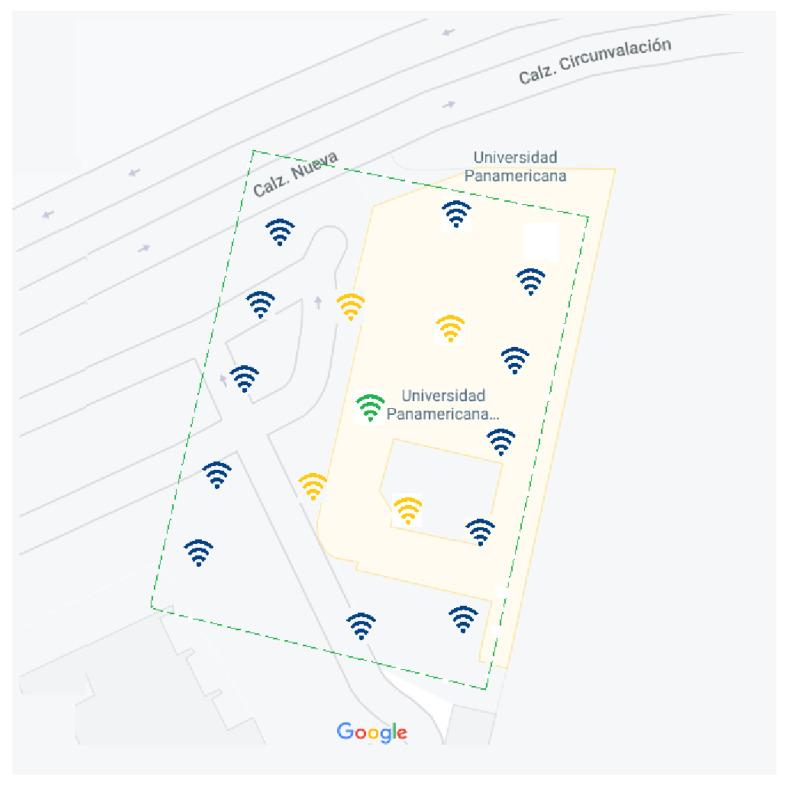
WSN location on campus within an area of 500 m${}^{2}$.

In addition to the simulation, and in order to complement the results of the simulation, measurements will be made from a WSN located in the engineering building of the Universidad Panamericana, Campus Guadalajara. Figure 5 presents a Google Maps search of the area where the WSN is physically installed and a general distribution of the sensors inside the network. Blue nodes are sensor nodes, while the green node is the coordinator and yellow nodes represent the possible jammer nodes. The network resembles the characteristics of an IWSN since in the area there is traffic of people using various mobile devices, cars in constant motion, school laboratories with electrical and computer machines as well as kitchens and offices with ventilation systems and computer equipment.

**Figure 6:**
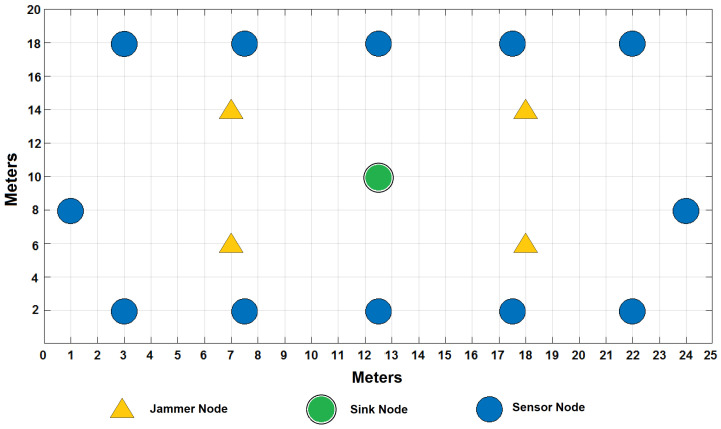
Distribution of the WSN nodes of the real experiment.

In the environment, there is a considerable traffic of people from Monday to Friday, since machines are being used in laboratories and personnel are working in their offices; traffic decreases considerably starting Saturday afternoon and all through Sunday as the campus closes its doors. Figure 6 shows the distribution of the WSN in a grid that covers 500 m${}^{2}$ of the campus in which, as already mentioned, signals of various types coexist, since it consists of different elements such as an avenue in the northern part where many types of vehicles circulate, in addition to some laboratories with industrial equipment, people circulating, gardens, parking lots, classrooms, etc. The experiment ran for a full seven days, starting on a Monday and ending on a Sunday with the sensors running under the same parameters from Table 4 that were used for simulations.

The network consists of 12 static and homogeneous nodes with sensors of various types sending information to the sink node, which is located in the center of the grid; four jammer nodes have also been placed, which will help to demonstrate the theme of this publication by seeing their impact on the cooperation and collaboration of the nodes to send the messages to the sink node in the best way. Six nodes are based on Zigbee wireless technologies and the other six are based on Lora. Texas Instruments CC2650 SimpleLink model, for sensor nodes, and CC2530 model, for gateway, have been used for the experiment. CC2650 SimpleLink supports Multi-Standard Wireless MCU: Bluetooth, Zigbee, 6LowPAN and IPv6, and offers low-power consumption supporting low-power sensors such as ambient light, infrared temperature, ambient temperature, accelerometer, gyroscope, magnetometer, pressure, humidity, microphone, magnetic sensor, etc. On the other hand, CC2530 is good in system-on-chip solutions for Zigbee and 2.4 GHz IEEE 802.15.4, optimum for industrial control and monitoring, building automation and low-power WSNs. Some other network parameters for the experiments are summarized in Table 6.

We want to test the experiment with a methodology in which, according to the PDR, RSSI and Energy performance metrics (which are simple, easy to detect and inexpensive), the network can manifest an anomalous behavior (symptoms) where it can tell that you are being attacked by a jammer or potential jammer. With the experiment, we are showing that our detection method does work in a Campus that has characteristics similar to an IWSN, with industrial protocols such as Zigbee and Lora, into a considerable area. We want to validate our algorithm and interference detection mechanism with the two technologies, not only with simulations but in a real environment. The following section will show the results obtained in the simulations and the experiment for each type of metric within the cooperative and collaborative network schemes.

## 4. Results

The results obtained from the simulations and the experiment are shown below:

### 4.1. Simulation 1

**Figure 7:**
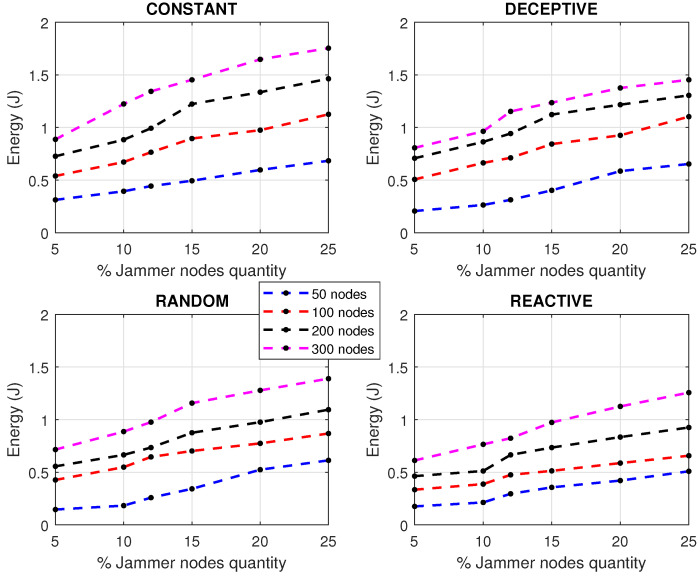
Types of jamming varying the number of nodes.

Increasing the number of jammer nodes in the network was used to evaluate and confirm the impact of jamming and different network densities in energy. In the simulation, we varied the quantity of jammer nodes present in the network, and the energy level variation of the network could thus be obtained for different types of jamming. Figure 7 presents the different results that were found when increasing the amount of nodes giving jamming interference to the network. When faced with networks with different numbers of nodes, the behavior they present when the number of jammer nodes increases is an almost linear increase in energy consumption.

Deceptive and constant jammers are those who make the network spend more energy in a steady state. When a constant jammer is present, it sends chaotic bits to the medium using no specific rules, so the communication is impaired by the attack. This excessive activity needs to use more processors and transmitters, and energy is wasted since instead of transmitting data, it is used to boycott the network. This type of attack is easier to detect due to the high impact it has on energy consumption. In a very different scenario, we find the reactive jammer, which is a spy who is always listening to the network waiting for the perfect moment to carry out an attack. When communication starts, this jammer corrupts the packets that are being sent by the sensor nodes at that moment. With reactive attack, the network spends on average less than 40% of the energy that a constant jammer would use to attack the network. The reactive jammer is always spying on the nodes, but the network suffers less than other types of attack because the nodes only lose power and packets at a specific time when the activity increases; notwithstanding, the foregoing, reactive jamming turns out to be in proportion to the time it acts, more efficient than other types of jammer.

**Figure 8:**
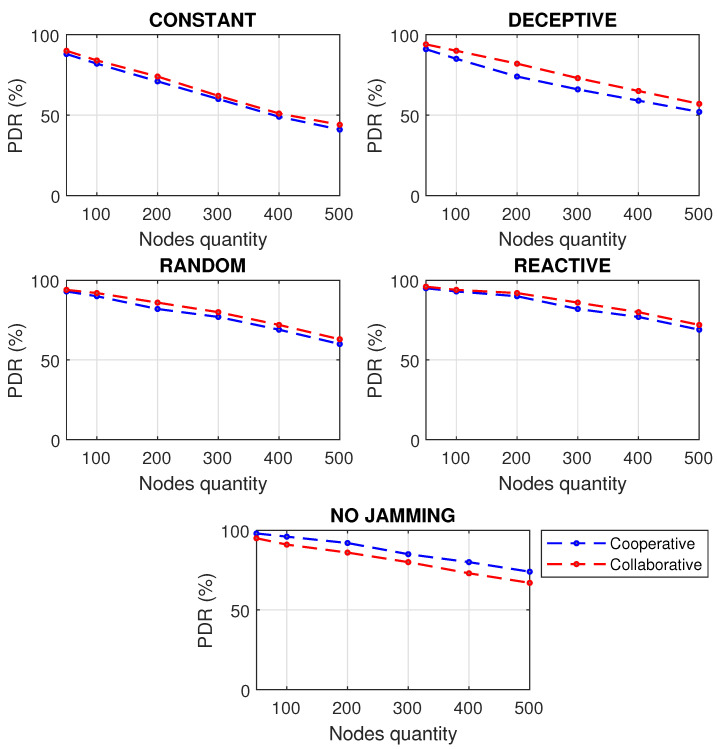
PDR for types of jamming in a WSN of 500 m${}^{2}$ with different number of nodes under collaborative and cooperative schemes.

It is important to note the relevance of detecting jamming attacks before their possible propagation to other nodes, since allowing the interference to be maintained, especially if 25% of the infected nodes were reached, in any of the scenarios shown, generates possible processor failures, packet loss, slow communication and a general state of instability. A bad state in the network will affect the correct monitoring of the industrial process causing economic losses. In addition to the above, results were obtained by performance metrics of the network subjected to various types of jamming, taking into account the approaches for cooperative and collaborative communication of the members of the network. First, as seen in Figure 8, the PDR simulation was carried out for cooperative and collaborative communication approaches, increasing the number of nodes to see the behavior of the network.

The network performs best under a cooperative scheme when it is not jammed; however, the collaborative scheme has better results when the network is faced with any kind of jamming interference. When there is no jamming, in the cooperative scheme, the nodes first process those activities that the network asks of them, and this path is more efficient because operations are centralized. However, at the time of a jamming attack, the network asks the node for incongruous activities because the communication is affected by the presence of the jammer, and the centralized communication no longer works, but it should be decentralized so that the nodes first process their information and then what the network asks of them. The absence of jamming means an optimal PDR, affecting more packet delivery in constant-type jamming, since the jammer is always decreasing the communication; even in reactive jamming, a small decrease in PDR can be perceived. It can be seen that since a WSN has more nodes, the difference between collaborative and cooperative schemes is more significant.

**Figure 9:**
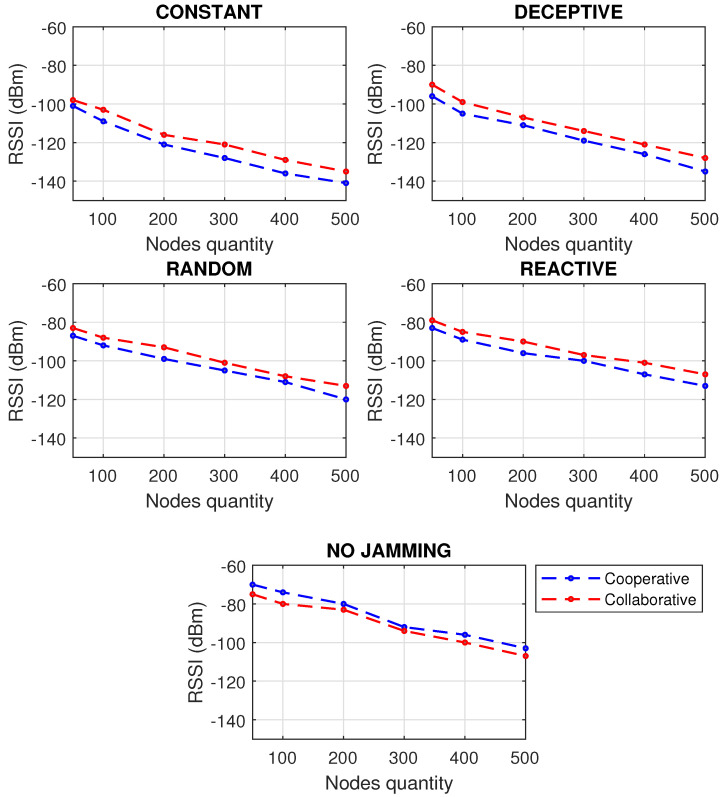
RSSI for types of jamming in a WSN of 500 m${}^{2}$ with different number of nodes under collaborative and cooperative schemes.

A similar analysis was carried out in Figure 9, for the RSSI, discovering that reactive jamming is the one that shows the best behavior for the network, followed by random, and far apart from these, deceptive and constant jamming, which have a noticeable dBm increase, reaching very low coverage levels. Once again it was shown that when there is no jamming, the cooperative scheme is better, because the nodes obey the sink node, and when the signal is lost due to jamming, the nodes take longer to know what to do because they are caught off guard and perform more information processing rediscovering the network, taking more time to find a fast route than with fewer hops to get the packets.

**Figure 10:**
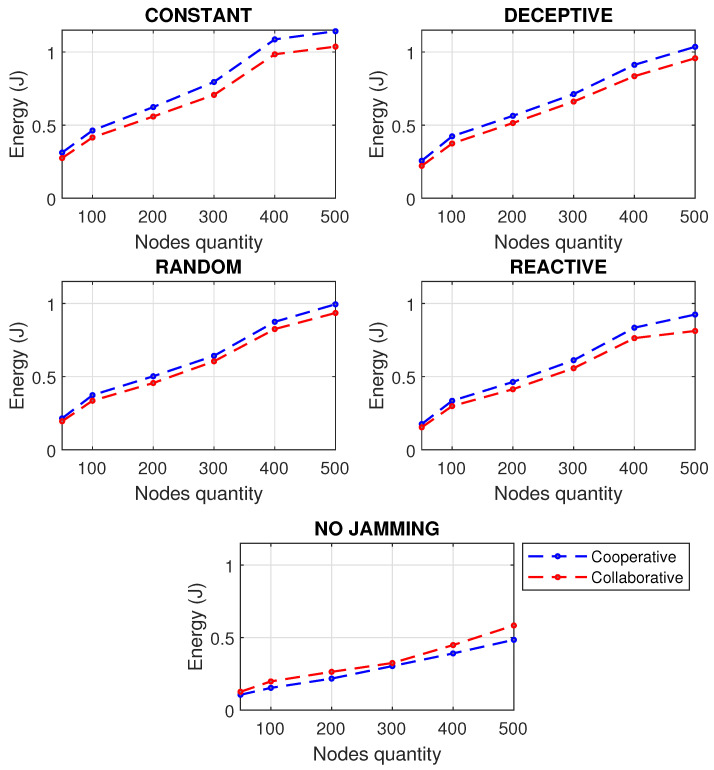
Energy for types of jamming in a WSN of 500 m${}^{2}$ with different number of nodes under collaborative and cooperative schemes.

PDR and RSSI have a significant impact on energy. Figure 10 shows that constant jamming is the one that makes the network consume more energy because it is the most demanding, while the reactive jamming takes less energy because it only attacks when it notices that there is activity on the network. As the number of nodes in a network increases, so does the energy that the cooperative scheme consumes with respect to the collaborative one.

The results obtained in the simulation of the performance metrics studied (PDR, RSSI and Energy) provide an approach to operating paradigms based on cooperation and collaboration, showing numerically an advantage of collaborative networks only when the network is experiencing a jamming attack: 4.9% improvement in RSSI metric when using a collaborative rather than cooperative network approach when being attacked by jamming; 4.4% improvement in PDR metric when using a collaborative network approach instead of cooperative when being attacked by jamming; and 10.7% energy is saved when using a collaborative rather than cooperative network approach when jammed.

### 4.2. Simulation 2

In the second simulation, it was decided to take a network of 200 nodes to increase the area and thus change the density to notice changes in the metrics and have other insights on the types of jamming and network schemes. For all the metrics, a sharp drop in their values was noted as the area of the network increased, approximately from 1000 m${}^{2}$ which is when the network began to decrease in density to a great extent.

**Figure 11:**
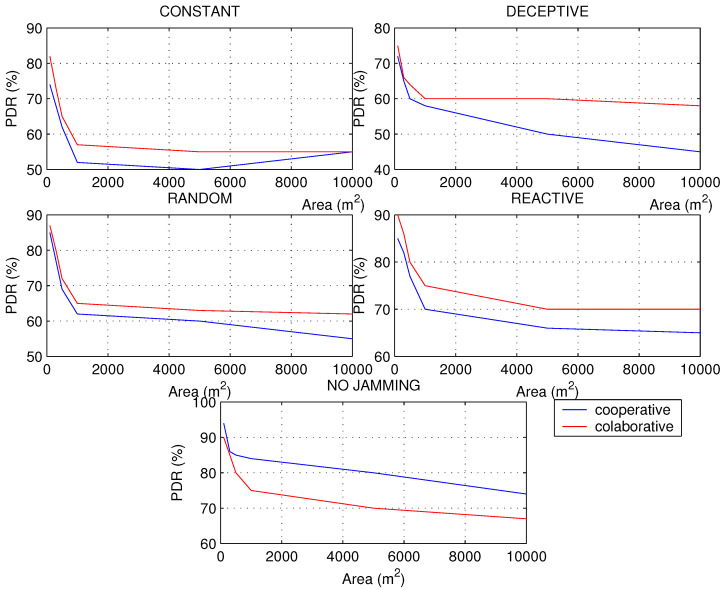
PDR for types of jamming in a WSN of 200 nodes with different areas under collaborative and cooperative schemes.

Networks with a higher density of nodes have a more predictable behavior than networks with a low density, since the scheme preference to be used in the processing algorithm can sometimes change. PDR has been calculated in Figure 11, seeing a greater aggressiveness of the constant jamming. The good response of the collaborative network is notable for networks with low node density and subject to deceptive interference. This response occurs because deceptive jamming proposes false information through interrupts, but they are not served because the node is configured to perform synchronous tasks, ignoring the deceptive targets that the jammer displays. However, when the network is in normal conditions, the cooperative scheme is more convenient, since there is no interference; waiting for the polling cycles generates a higher latency than the interruptions proposed by the cooperative scheme.

In networks with high node density, jamming manifests itself with a rapid drop in RSSI, especially in deceptive jamming; however, when node density decreases, increasing the area of distribution of nodes, reactive jamming shows more aggressiveness. This is because of the fact that when the distances are greater between the nodes, there is room for greater interferences than in small areas, which are usually more controlled.

**Figure 12:**
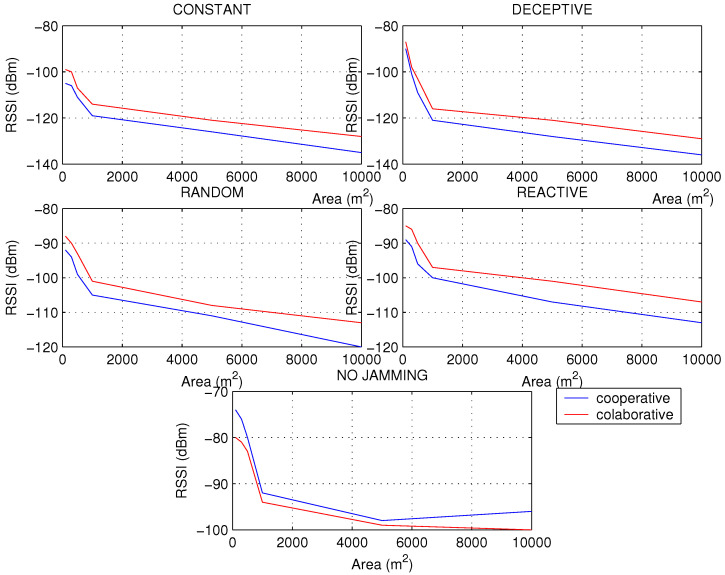
RSSI for types of jamming in a WSN of 200 nodes with different areas under collaborative and cooperative schemes.

Figure 12 shows that before jamming attacks, the RSSI will always decrease, but under normal conditions a curious phenomenon can occur, and it is the sudden increase in the RSSI in networks that are not very dense, especially for cooperative schemes. This makes sense considering that there should be fewer packet collisions as there is more space for transmission.

**Figure 13:**
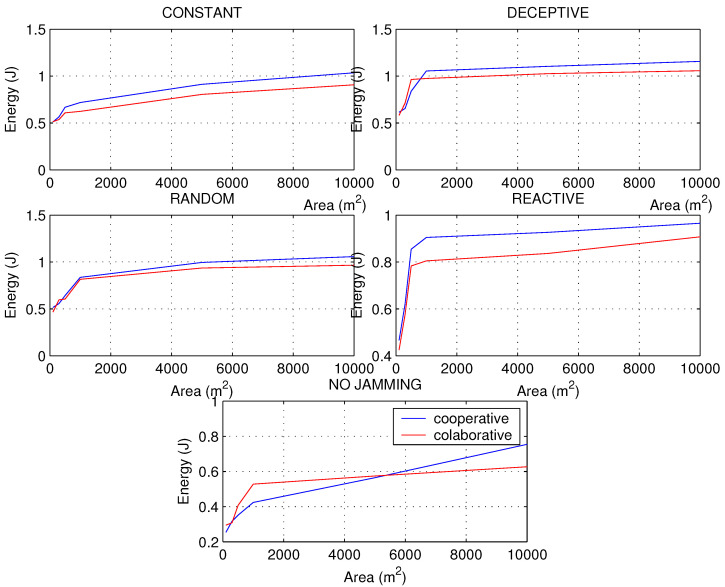
Energy for types of jamming in a WSN of 200 nodes with different areas under collaborative and cooperative schemes.

Regarding the energy consumption of the nodes, we know that the PDR has a strong impact on the consumption level, so that if the constant and random jamming caused a strong drop in the PDR of the network, it will also cause it in the energy, but more constantly, without resounding drops, as occurs in reactive jamming in networks with a higher density of nodes. It is observed in Figure 13 where jamming attacks the cooperative network, causing it to generate more retransmissions and CPU usage to find the way with the best LQI to send the packets through the best route in less time; all this translates into higher energy consumption. As in the RSSI, it is observed that after 5000 nodes, when the density of nodes decreases, the cooperative scheme has a considerable improvement over the collaborative one, so it is recommended to use the cooperative scheme in networks of low node density only when there is no interference. The energy consumption shows the damage caused by random jamming in terms of the effectiveness of the aggression per unit of time, especially in the cooperative scheme, since this type of jammer makes a combination of constant attack with reactive attack.

The second simulation has been very helpful to broaden the perspective of the behavior of networks under different densities of nodes, since in practical applications such as IWSN, where there is a high *density*, or applications in the field such as in agriculture or livestock monitoring, they can now have recommendations for detecting jamming problems adapted to each use case.

### 4.3. Experiment

To strengthen the results of the simulations, the experiment was carried out with the WSN on campus, achieving performance metrics levels in steady state so that the quality of communication between the nodes of the network can be known. The above can be very useful in any other environment where it is implemented, such as industrial environments. Table 7 shows the stationary values obtained by the experiment network, which have served to validate through a second iteration the proposed algorithm presented in Figure 4.

Given the appearance of jamming, there is a constant better performance when a collaborative scheme is used, and what the simulations had shown is demonstrated, i.e., when there is no jamming, the cooperative scheme presents better metrics. It is important to remember that it is convenient to use the algorithm proposed in networks of medium to high density of nodes so that it has an accurate effect, since the simulations and experiment with which it was validated are focused on places that meet the same characteristics that are observed in warehouses and smart SMEs. The existing intensity in the communication channel, provided by the RSSI, can come from various sources such as transmitters, jammers, radiation, etc., giving us a level of energy detection that will help the nodes to decide the routes to take in the shipment of packages.

**Figure 14:**
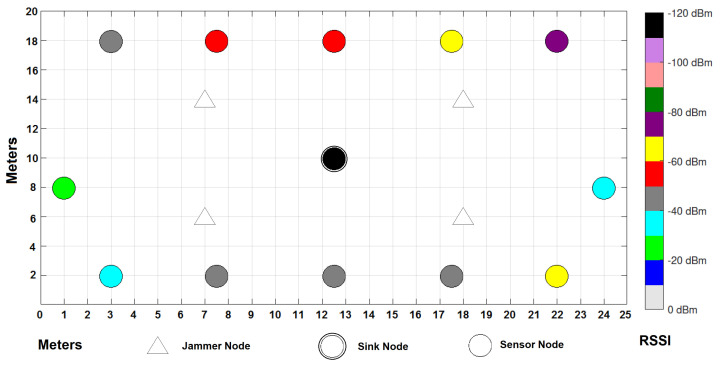
RSSI results per node under a cooperative scheme.

**Figure 15:**
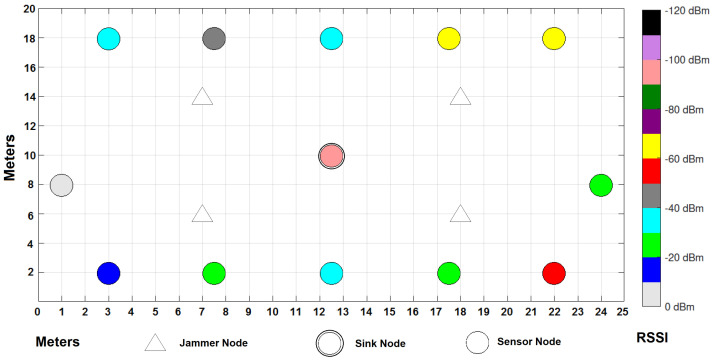
RSSI results per node under a collaborative scheme.

As can be seen in Figure 14 and Figure 15, the collaborative approach presents better levels of RSSI than the cooperative approach. There is a node where the signal strength is almost zero when applying a collaborative approach to communication between sensors. It can also be observed that the coordinator presents a better RSSI in the collaborative approach (−100 dBm) than in the cooperative approach (−120 dBm).

**Figure 16:**
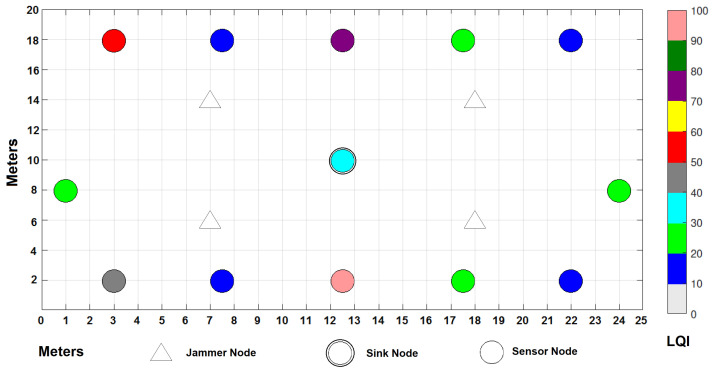
LQI results per node under a cooperative scheme.

**Figure 17:**
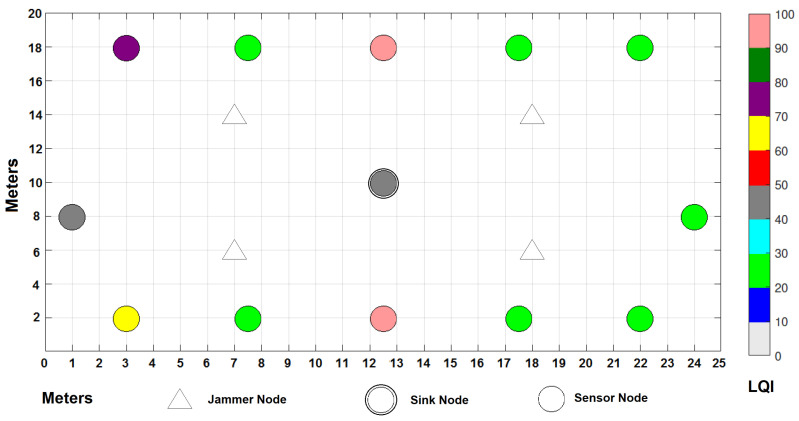
LQI results per node under a collaborative scheme.

In addition to RSSI, LQI was measured in the experiment. The quality of reception of data packets is provided by the LQI, which, together with energy efficiency, is an important element when deciding which route a message should take. LQI and RSSI always go hand in hand and add value by providing shorter Round Trip Time (RTT) for packet travel from transmitter to final receiver. As can be seen in Figure 16 and Figure 17, the LQI improves under a collaborative scheme, because the node processor seeks to save its own energy first, which makes it choose to send the packets through the routes where there is a higher level of LQI, thus decreasing latency and influencing the decrease in energy consumption.

In practice, since the sniffer of the sensors allows us to know these two parameters in each sensor, it is possible to relate them; since packet loss is mitigated by re-transmitting packets on those routes with better LQI and RSSI, these routes serve as a useful means of reduced latency and power distribution.

In the next subsection, a validation of the jamming detection algorithm is performed using data obtained from the experiment.

### 4.4. Jamming Detection Algorithm Use Case

For this example, the steps of the algorithm for the detection of jamming indicated in Figure 4 have been followed. The example takes the results of the experiment carried out that are found in Table 7. The selected initial setup is done in the first four steps:

Step 1: Select network scheme: cooperative.
Step 2: Select number of nodes: 12.
Step 3: Select area: 500 m${}^{2}$.
Step 4: Obtain *data* and *data labels* (Table 8) from tables that were built with the results of the experiment (Table 7).

Next, the steady-state values of the performance metrics presented by the WSN nodes are obtained.

Step 5: Detect signal in real time.
Step 6: Steady-state WSN metrics:
  Measure Energy: Data 1 = 0.265 J
  Calculate PDR: Data 2 = 95.5%
  Calculate RSSI: Data 3 = −86 dBm
Step 7: Initialize Jamming Counters: $C_{C_{O}}=0$, $C_{D_{E}}=0$, $C_{R_{A}}=0$, $C_{R_{E}}=0$ and $C_{N_{J}}=0$.
Step 8: Sort data from smallest (A) to largest (E) (see Table 9).

Step 9: Determine decision threshold values $Th_{A_{B}n}$, $Th_{B_{C}n}$, $Th_{C_{D}n}$, $Th_{D_{E}n}$ (where *n* = 1 for Energy, *n* = 2 for PDR, and *n* = 3 for RSSI) by averaging the immediate upper and lower data values (see Table 10). For example:
  (5) $$\begin{array}{c} Th_{A_{B}1}=\left[\frac{A+B}{2}\right]=\left[\frac{0.09+0.2}{2}\right]=0.145 \end{array}$$

Step 10: *n* = 1.

Energy analysis:

Step 11: (*n* = 1): Data = Data 1, where Data 1 = 0.265 J (from step 6).

(Steps 12 and 13 are presented after energy analysis as they repeat Steps from 14 to 32).

**Figure 18:**
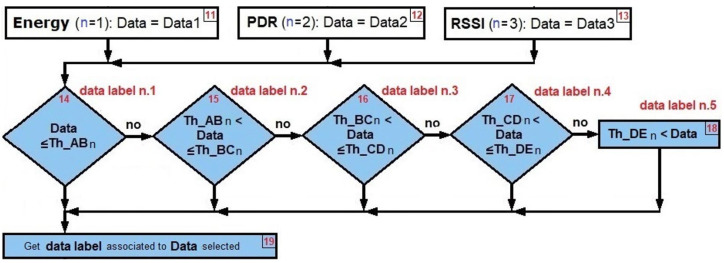
*Data threshold* decisions.

Rhombs from Figure 18 are used to decide from steps 14 to 19:

Step 14: First rhomb: Data 1 (0.265) $<=Th_{A_{B}1}$ (0.145) ⇒no.

Step 15: Second rhomb $Th_{A_{B}1}\left(0.145\right)<Data1\left(0.265\right)<=Th_{B_{C}1}\left(0.23\right)$ ⇒no.

Step 16: Third rhomb $Th_{B_{C}1}\left(0.23\right)<Data1\left(0.265\right)<=Th_{C_{D}1}\left(0.295\right)$ ⇒yes.

Step 17: Fourth rhomb $Th_{C_{D}1}\left(0.295\right)<Data1\left(0.265\right)<=Th_{D_{E}1}\left(0.35\right)$ ⇒no.

**Figure 19:**
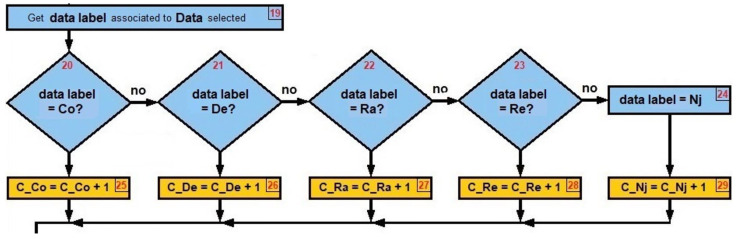
*Data label* decisions.

Step 18: $Th_{D_{E}}\left(0.35\right)<Data1\left(0.265\right)$ ⇒no, then:

Step 19: Obtain *data label* associated to Data selected (see Figure 19).

Step 20: Is *data label* = $C_{O}$? ⇒no.

Step 21: Is *data label* = $D_{E}$? ⇒no.

Step 22: Is *data label* = $R_{A}$? ⇒yes.

Step 23: Is *data label* = $R_{E}$? ⇒no.

Step 24: Is *data label* = $N_{J}$? ⇒no.

Step 25: $C_{C_{O}}=0$.

Step 26: $C_{D_{E}}=0$.

Step 27: $C_{R_{A}}=0+1=1$.

Step 28: $C_{R_{E}}=0$.

Step 29: $C_{N_{J}}=0$.

Step 30: *n* = *n* + 1 = 1 + 1 = 2.

Step 31: RSSI analyzed (n>3)?⇒no.

Step 32: PDR analyzed (n>2)?⇒no.

PDR analysis: Next, steps 14 to 32 are repeated, but this time *n* = 2, so the PDR is now analyzed.

Step 12: (*n* = 2): Data = Data 2, where Data 2 = 95.5 % (from step 6).

Rhombs from Figure 18 are used to decide from steps 14 to 19 again:

Step 14: First rhomb: Data 2 (95.5) $<=Th_{A_{B}2}$ (94.5) ⇒no.

Step 15: Second rhomb $Th_{A_{B}2}\left(94.5\right)<Data2\left(95.5\right)<=Th_{B_{C}2}\left(95\right)$ ⇒no.

Step 16: Third rhomb $Th_{B_{C}2}\left(95\right)<Data2\left(95.5\right)<=Th_{C_{D}2}\left(96\right)$ ⇒yes.

Step 17: Fourth rhomb $Th_{C_{D}2}\left(96\right)<Data2\left(95.5\right)<=Th_{D_{E}2}\left(98\right)$ ⇒no.

Step 18: $Th_{D_{E}2}\left(98\right)<Data2\left(95.5\right)$ ⇒no, then:

Step 19: Obtain *data label* associated to Data selected (see Figure 19).

Step 20: Is *data label* = $C_{O}$? ⇒no.

Step 21: Is *data label* = $D_{E}$? ⇒no.

Step 22: Is *data label* = $R_{A}$? ⇒yes.

Step 23: Is *data label* = $R_{E}$? ⇒no.

Step 24: Is *data label* = $N_{J}$? ⇒no.

Step 25: $C_{C_{O}}=0$.

Step 26: $C_{D_{E}}=0$.

Step 27: $C_{R_{A}}=1+1=2$.

Step 28: $C_{R_{E}}=0$.

Step 29: $C_{N_{J}}=0$.

Step 30: *n* = *n* + 1 = 2 + 1 = 3.

Step 31: RSSI analyzed (n>3)?⇒no.

Step 32: PDR analyzed (n>2)?⇒yes.

RSSI analysis: Next, steps 14 to 32 are repeated, but this time *n* = 3, so the RSSI is now analyzed.

Step 13: (*n* = 3): Data = Data 3, where Data 3 = −86 dBm (from step 6).

Rhombs from Figure 18 are used to decide from steps 14 to 19 again:

Step 14: First rhomb: Data 3 (−86) $<=Th_{A_{B}3}$ (−102.5) ⇒no.

Step 15: Second rhomb $Th_{A_{B}3}\left(-102.5\right)<Data3\left(-86\right)<=Th_{B_{C}3}\left(-94.5\right)$ ⇒no.

Step 16: Third rhomb $Th_{B_{C}3}\left(-94.5\right)<Data3\left(-86\right)<=Th_{C_{D}3}\left(-85\right)$ ⇒yes.

Step 17: Fourth rhomb $Th_{C_{D}3}\left(-85\right)<Data3\left(-86\right)<=Th_{D_{E}3}\left(-76.5\right)$ ⇒no.

Step 18: $Th_{D_{E}3}\left(-76.5\right)<Data3\left(-86\right)$ ⇒no, then:

Step 19: Obtain *data label* associated to Data selected (see Figure 19).

Step 20: Is *data label* = $C_{O}$? ⇒no.

Step 21: Is *data label* = $D_{E}$? ⇒no.

Step 22: Is *data label* = $R_{A}$? ⇒yes.

Step 23: Is *data label* = $R_{E}$? ⇒no.

Step 24: Is *data label* = $N_{J}$? ⇒no.

Step 25: $C_{C_{O}}=0$.

Step 26: $C_{D_{E}}=0$.

Step 27: $C_{R_{A}}=2+1=3$.

Step 28: $C_{R_{E}}=0$.

Step 29: $C_{N_{J}}=0$.

Step 30: *n* = *n* + 1 = 3 + 1 = 4.

Step 31: RSSI analyzed (n>3)?⇒yes.

Step 32: *Does not apply as (n > 3)*.

**Figure 20:**
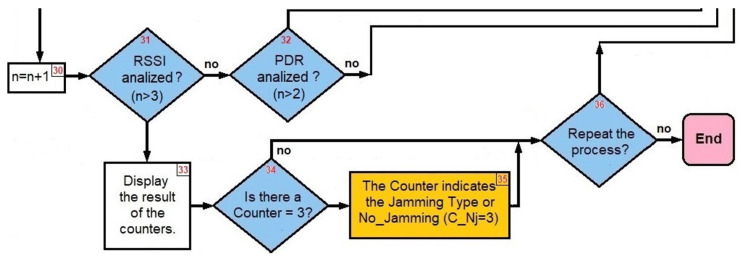
Final steps.

Once the three performance metrics have been analyzed, we move on to the final part of the algorithm (Figure 20).

In this example, the following results are obtained:

Step 33: Display the result of the counters: $C_{C_{O}}=0$, $C_{D_{E}}=0$, $C_{R_{A}}=3$, $C_{R_{E}}=0$ and $C_{N_{J}}=0$.

Step 34: Is there a Counter =3? ⇒yes.

Step 35: Because $C_{R_{A}}=3$ then there is *Random Jammer* in the network.

Step 36: Repeat the process? ⇒no.

End.

The proposed mechanism for the detection of jamming has been validated; thanks to these results, we can extrapolate jamming detection to other types of industries such as maintenance and smart manufacturing. The lessons learned, as well as their impact on safety and reduction of operating and maintenance costs, are discussed below.

## 5. Discussion

The results have allowed us to obtain the elements to answer the research question: *Which configuration helps more to protect a WSN from jamming?* The answer is that while a cooperative approach leads to a normally functioning network, a collaborative approach offers greater protection to a network when there is jamming, since each node ensures the transmission of its own information before committing to the network. Collaboration helps decrease collisions and retries, making the node’s processor consume less power. It is important to note that this result was obtained for all types of jamming, so the collaborative scheme gives us to think about possibilities to propose a fast jamming mitigation algorithm.

**Aggressor or type of jamming attack**. The *constant* jammer showed to be the most scandalous in terms of the change in metrics in all types of jamming. It is the jammer that causes more collisions, and this was reflected in a decrease in the PDR and the RSSI, and an increase in energy; it is a type of jamming easier to detect since it emits random bits, which no node would take as valid but is rejected more easily. On the other hand, *deceptive* jammer had results very similar to the constant, only with slightly less aggressive levels, since while the constant jammer always emits random bits, the deceptive jammer consumes more time in processing data sending because it continuously emits legitimate packets, which reach other nodes believing that they contain valid information. Regarding *random* jamming, it can have a trade-off in that it is a little more mechanical to process it at the algorithm level; however, it has a high rate of assertiveness in terms of causing collisions, more than deceptive and constant. Finally, *reactive* is the most aggressive jamming because it has a nature, which has a greater expense on the part of the jammer node but has a more aggressive nature in the sense that the node is always aware of any communication, so it does not allow even the formation of a network. This jamming is the most aggressive, but it is the one that has the best energy performance for the aggressor node. In an IWSN, the reactive jamming is the one that can cause the greatest damage to machines in a short time, so we can use mechanisms to prevent it and not cause loss of money and time in the production.

When jamming is more aggressive, such as reactive and random, a phenomenon begins to occur that collisions begin to be so large that a series of packet retransmissions is triggered, which in turn lead to failed attempts to listen to the channel, excessive expenditure of energy, and therefore by constantly listening to the channel in an unsatisfactory way. The expense that is generated in the processing algorithm and in the delay that is run in this processing algorithm listens to the channel, which is specifically the CSMA/CA protocol, and leads to a deterioration of the quality of the information in the network. The delays begin to be so great that the number of error packets, obsolete routes, or lost routes in the network begins to increase; the above creates an overhead, an overload in the network that will lead to the same collisions, the loss of routes and obsolescence of the same (of the routes) which is a technique that makes the reconfigurations in the network change unnecessarily, because if there is the obsolescence of routes the nodes are reconfigured, the topology is reconfigured, and this causes a false alarm regarding the routes and an energy expenditure, and this generates delays, and losses of information in WSN configuration.

**Density**. Regardless of whether or not a jamming attack exists, as well as regardless of whether a collaborative or cooperative scheme is being used, it has been shown that by increasing the number of nodes and leaving the fixed area of 500 m${}^{2}$ (an increase in density), the distances between the nodes decrease, and the symptoms that make us realize this are a decrease in the PDR and the RSSI, and an increase in the energy consumed by the node. This is due to shorter distances between nodes, less space for packet transmission, and increased collisions. When there are more collisions, fewer packets reach their destination, and this is observed in the decrease in the PDR. As noted in Equation (3), it is known that the distance between the sender and the receiver is directly proportional to the energy received and inversely proportional to the RSSI. Notwithstanding all the above, by increasing the coverage area leaving the number of nodes fixed (an increase in density), it was found that everything mentioned above can only be valid for networks with areas less than approximately 1000 m${}^{2}$, since with larger areas the behavior is reversed, having for little dense networks a decrease in the PDR, in the RSSI and an increase in the Energy. This makes sense since when data is transmitted at distant distances, there are atmospheric phenomena, noises typical of the environment where the WSN is installed; if it were an IWSN with very large distances, communication could collapse due to the excess of failed attempts to listen to the channel due to the unintentional noises caused by the machines. Both in the simulations and the experiment, it was found that taking the density of nodes as a parameter for detection can give us a guideline for their future mitigation, contributing to the security of the WSN.

**Network immunity**. The collaborative scheme is more resilient to jamming attacks because, although it has a high-power consumption because its processing protocol works harder when a jamming attack occurs, the environment around it is the one that changes, and the previously high energy level is now lower than the rest of the environment found in overhead. The change in the metrics of a cooperative network in the face of an attack of any type of jamming is much greater than the change presented by a collaborative network. According to the results, it can be said that a collaborative protocol offers greater immunity to jamming attacks but consumes more energy. On the other hand, the collaborative scheme maintained better performance in both low-density networks and high-density networks of nodes, which made it ideal for aggressive environments, such as a factory. Everything to be done lays the groundwork for considering these schemes in a jamming mitigation strategy based on a situational routing protocol, which behaves collaboratively under normal conditions but can be collaborative in the face of jamming attacks. This opens a new way of looking at the security management of a WSN.

**Symptoms or jamming metrics**. According to the results, we can say the following about the metrics or symptoms: First, the *PDR* tells us how reliable a node is, so keeping its level high is important. It is a metric based on the results of packets delivered, which makes it optimal to know how secure a network is. This metric can have bad levels not only due to the presence of jamming, but also when there are many collisions in the environment (high-density networks), or when there are failures in the nodes of the neighbors (when they are too far away), or when there are imperfect connections. Secondly, the *RSSI* maintained at good levels helps us to make the network available to transmit at any time. The health status of the communication channel is measured by this metric. The RSSI translates into availability because the RSSI is affected by the loss of routes, which is when the power in space of the electromagnetic wave that goes from the transmitter to the receiver decreases. In addition, RSSI is also affected by fading, which is the deviation from the attenuation experienced by a signal, as well as by shading, which is when the signal is lost by surrounding objects or people. Finally, the *Energy* is a symptom that is easier to quantify in monetary costs for a company, and for this reason, it is important to measure it and know which are the independent variables that affect it in a greater way. The jamming metrics used can be complemented by other metrics such as SNR or BER, so that the proposed algorithm can be extended for networks with lower densities.

The *detection algorithm* has laid the foundations for the mitigation of jamming since the damage caused to the network by each type of jamming has been detected, and it has been shown that in higher density networks, its detection is better.

## 6. Conclusions

The configuration that helps to protect a WSN the most is a combination of elements that are summarized in the *jamming detection mechanism* proposed in this work.

To better protect a WSN, it is important to know the ways it is attacked. With the chosen configurations, *constant* jamming was the one that most affected the PDR and RSSI of the network. In networks with very wide coverage areas, it was observed that the *deceptive* jammer did not damage the network much since long distances also lost many of the legitimate packets sent by the jammer. On the other hand, *reactive* jamming is the most effective since in the short time it acts, it considerably damages the WSN, making the network consume more energy especially in networks with a wide coverage area. The damage that the reactive jammer causes is remarkable, consuming less than half the energy that it would cause if it were a constant or deceptive jammer. Finally, the *random* jammer toggles between sleep mode and the emission of random and legitimate bits, saving energy, which makes it useful for nodes with short battery life. Although it does not damage the network as much as a reactive jammer, this type of jamming accomplishes its objective using few resources.

The practical applications of WSN can be classified according to their *density*, being those with more node density in which more abrupt changes in performance metrics occur. The results demonstrated that the proposed algorithm works for areas of medium to high node density, such as smart factory applications. It is recommended to implementing a cooperative approach when both the number of nodes and coverage is small, whereas a collaborative approach is better when the number of nodes increases in the same area. When there are many nodes, packet collisions increase, making it more useful for each node to make sure to process its information before helping the network.

The collaborative approach is more resilient than the cooperative to jamming attacks. The simulations and the experiment showed the conditions in which a network scheme can offer greater immunity to the network. In general, and especially in projects that are scalable in number of nodes, it has been shown that it is convenient to use a collaborative configuration, which provides better results in performance metrics. When there is no jamming attack, it is more convenient to preserve the cooperative paradigm since it is easier to configure because one node is the one that maintains the leadership in communication, but when the network is subject to a jamming attack, the entire communication can go down if a collaborative reaction strategy is not planned to help maintain communication between nodes optimally.

The energy savings provided by the collaborative configuration in the face of jamming would have a significant impact on industrial maintenance costs since machine sensors are the inputs that a machine has to obtain data from the world and use for its purpose. To achieve quantitative results of this, it will be necessary to involve industrial maintenance metrics. Jamming detection based on performance metrics such as PDR and RSSI helps increase network *reliability* and *availability* in IWSN. Knowing and detecting the types of jamming will go a long way in keeping production systems up and running in factories, and their rapid detection and mitigation save businesses time and money.

A novel element in WSNs is to use performance metrics at the network layer level [59,60]. Thanks to these metrics, we can propose future works for the improvement of the administration and optimization of the routing protocols. We want to reach a point where the routing protocol can propose the *symptoms* of jamming and can react through a *mitigation proposal*. This mitigation proposal must use cooperative or collaborative schemes in a situational way. To strengthen this situational mitigation, it will be considered that as the nodes that make up a WSN are intelligent and therefore learn from what happens in the environment in which they operate, it is therefore convenient to adapt some of the learning theories to communication between the nodes of a WSN, such as collaborative, cooperative, environmental and knowledge-based learning [61]. In future work, other schemes for wireless network communications will be proposed. These possible approaches could have positive contributions in the application of WSN to factories, improving safety and reducing operating and maintenance costs.

## Figures and Tables

**Table 1:** Types of jamming attacks.

Types of Jammers			Saving of Energy	Proactive (P) or Reactive (R)
Elementary		Constant		P
	Proactive	Deceptive		P
		Random	✓	P
	Reactive	Data/Ack (acknowledge)		R
		Request to send (RTS)/Clear to send (CTS)		R
Advanced		Control Channel	✓	P and R
	Smart Hybrid	Implicit	✓	P and R
		Flow	✓	P and R
	Function-Specific	Follow On	✓	P
		Channel Hopping		R
		Pulsed Noise		P

**Table 2:** Overview of detection techniques.

Detection Technique	Metrics	Description
PDR with consistency checks [31]	PDR, packet sending ratio (PSR) and sensing time	Low PDR leads to the detection of jamming. Consistency checks are used.
Exponentially weighted moving average (EWMA) [20]	Inter arrival time (IAT)	Statistical method using inter arrival times of packets.
Fuzzy interference system [14]	SNR: packet dropped per terminal	This is followed by a confirmatory check and a 2-means clustering of neighborhood nodes.
Ant system [32]	Hops, energy, distance, SNR, BER, PDR	The Ant collects data from various routes with which a destination is reached, indicating if there is a jammer.
Jammed-area mapping protocol [33]	Clear channel assessment (CCA)	The number of unsuccessful attempts to capture wireless channel is counted; if the count is greater than 10, a jammer is detected.
Channel surfing and spatial retreat [11]	CSMA, ambient noise levels	By measuring ambient noise levels, the detection is conducted at either MAC layer using CSMA or physical level.
Reactive detection [34]	BER, RSSI	The RSSI of each bit is observed on reception. A high RSSI means that there is a jammer.
Trigger nodes identification [35]	Curvilinear component analysis (CCA)	Techniques are integrated: the group testing, the disk cover and the clique-based clustering.
Fighting implicit jamming (FIJI) [36]	Delay throughput	While non-jammed, the clients are unaffected, but while being jammed, the clients receive maximum throughput.
Cross-layer system [37]	Frequency-hopping pattern	Detection is done when the transmitter uses additional test patterns during its transmission.
Control channel attack prevention [38]	Hamming distance	Is a cross layer system that implements part of the system network module and part in the driver.
Game theoretic modeling [39,40]	Request to send (RTS), network allocator value	Cluster retransmission of data using CSMA/CD (collision detection) and a network allocator value.

**Table 3:** Jamming *symptoms*.

Type of Jamming	PDR	RSSI	Energy	BPR	SNR	BER
Constant	−−	++	++	−	≤1	−
Deceptive	−−	++	++	+	≤1	−
Random	−	+	+	+	≤1	+−
Reactive	−	+	+	+	≤1	+−

Increases = “+”, increases a lot = “++”, decreases = “−”, decreases a lot = “−−”, oscillates = “+−”.

**Table 4:** Simulation parameters under CSMA/CA [55,56,57].

Parameter	Value
**Physical Layer Parameters**	
Sensitivity threshold	−85 dBm
Transmission power	0 dBm
**MAC Layer Parameters**	
Maximum retransmission number	3
Maximum retry number	5
Maximum number of tries to reach a node from the collector	9
Packet error rate (PER)	1% to 4%
Average frame length	22 bytes
Maximum number of back-offs	4
Packet frame size	30 bytes
MAC protocol	IEEE 802.15.4
MAC layer	CSMA/CA
**Network Layer Parameters**	
Maximum Bit Rate - Worldwide	250 kbps
Scenario	Static nodes
Operational frequency band	2.4 GHz (Worldwide ISM band)
**Simulation 1:**	
Network area	500 m${}^{2}$
Number of nodes	50, 100, 200, 300, 400 and 500
Sink location	Center
**Simulation 2:**	
Network area (m${}^{2}$)	100, 300, 500, 1000, 5000, 10,000
Number of nodes	200
Sink location	Center

**Table 5:** Energy model.

Node Task or Activity		Voltage (mV)	Current (mA)	Time (ms)	Energy (J)
$E_{S}$	Start-up mode	120	12	0.2	0.000288
$E_{M}$	MCU (32-MHz clock)	75	7.5	1.7	0.000956
$E_{CSMA}$	CSMA/CA algorithm	270	27	1.068	0.00778
$E_{SW1}$	*R_x_* to *T_x_* switching	140	14	0.2	0.000392
$E_{SW2}$	*T_x_* to *R_x_* switching	250	25	0.2	0.00125
$E_{R_{x}}$	Reception mode	250	25	4.1915	0.0262
$E_{T_{x}}$	Transmission mode	320	32	0.58	0.00426
$E_{SD}$	Shut down mode	75	7.5	2.5	0.00141

**Table 6:** Experiment parameters.

Parameter	Value
Experiment area	500 m${}^{2}$
Number of sensor nodes	12
Sink nodes	1
Topology	Grid
Node RAM	128 KB
Normal voltage range	1.8 V to 3.8 V
Bit architecture	16

**Table 7:** Experiment results.

Jamming	PDR (%)		RSSI (−dBm)		Energy (J)	
	**Cooperative**	**Collaborative**	**Cooperative**	**Collaborative**	**Cooperative**	**Collaborative**
Constant	94	96	−103	−98	0.37	0.32
Deceptive	95	96	−102	−92	0.33	0.29
Random	95	97	−87	−84	0.26	0.21
Reactive	97	98	−83	−80	0.2	0.13
No Jamming	99	97	−70	−75	0.09	0.07

**Table 8:** *Data* and *data labels*.

Metric or Symptom	*Data label*				
	$C_{O}$ (Constant)	$D_{E}$ (Deceptive)	$R_{A}$ (Random)	$R_{E}$ (Reactive)	$N_{J}$ (No Jamming)
Energy (J)	0.37	0.33	0.26	0.2	0.09
PDR (%)	94	95	95	97	99
RSSI (−dBm)	−103	−102	−87	−83	−70

**Table 9:** Sorted data from smallest (A) to largest (E).

	**A**	**B**	**C**	**D**	**E**
Energy (J)	$N_{J}$	$R_{E}$	$R_{A}$	$D_{E}$	$C_{O}$
	0.09	0.2	0.26	0.33	0.37
	**A**	**B**	**C**	**D**	**E**
PDR (%)	$N_{J}$	$R_{E}$	$R_{A}$	$D_{E}$	$C_{O}$
	94	95	95	97	99
	**A**	**B**	**C**	**D**	**E**
RSSI (−dBm)	$N_{J}$	$R_{E}$	$R_{A}$	$D_{E}$	$C_{O}$
	−103	−102	−87	−83	−70

**Table 10:** Decision threshold values.

	**A**	$Th_{A_{B}1}$	**B**	$Th_{B_{C}1}$	**C**	$Th_{C_{D}1}$	**D**	$Th_{D_{E}1}$	**E**
Energy (J)	$N_{J}$		$R_{E}$		$R_{A}$		$D_{E}$		$C_{O}$
	0.09	0.145	0.2	0.23	0.26	0.295	0.33	0.35	0.37
	**A**	$Th_{A_{B}2}$	**B**	$Th_{B_{C}2}$	**C**	$Th_{C_{D}2}$	**D**	$Th_{D_{E}2}$	**E**
PDR (%)	$N_{J}$		$R_{E}$		$R_{A}$		$D_{E}$		$C_{O}$
	94	94.5	95	95	95	96	97	98	99
	**A**	$Th_{A_{B}3}$	**B**	$Th_{B_{C}3}$	**C**	$Th_{C_{D}3}$	**D**	$Th_{D_{E}3}$	**E**
RSSI (−dBm)	$N_{J}$		$R_{E}$		$R_{A}$		$D_{E}$		$C_{O}$
	−103	−102.5	−102	−94.5	−87	−85	−83	−76.5	−70

## Data Availability

The data presented in this study are available on request from the corresponding author.
